# WBSMDA: Within and Between Score for MiRNA-Disease Association prediction

**DOI:** 10.1038/srep21106

**Published:** 2016-02-16

**Authors:** Xing Chen, Chenggang Clarence Yan, Xu Zhang, Zhu-Hong You, Lixi Deng, Ying Liu, Yongdong Zhang, Qionghai Dai

**Affiliations:** 1 National Center for Mathematics and Interdisciplinary Sciences, Chinese Academy of Sciences, Beijing, 100190, China; 2 Academy of Mathematics and Systems Science, Chinese Academy of Sciences, Beijing, 100190, China; 3 Institute of Information and Control, Hangzhou Dianzi University, Hangzhou, 310018, China; 4 Department of Automation, Tsinghua University, Beijing, 100084, China; 5 School of Mechanical, Electrical & Information Engineering, Shandong University, Weihai, 264209, China; 6 School of Computer Science and Technology, China University of Mining and Technology, Xuzhou, 221116, China; 7 Institute of Computing Technology, Chinese Academy of Sciences, Beijing, 100190, China; 8 University of Chinese Academy of Sciences, Beijing, 100049, China; 9 School of Economics and Management, Beihang University, Beijing, 100191, China; 10 Key Lab of Intelligent Information Processing of Chinese Academy of Sciences, Institute of Computing Technology, Chinese Academy of Sciences, Beijing, 100190, China

## Abstract

Increasing evidences have indicated that microRNAs (miRNAs) are functionally associated with the development and progression of various complex human diseases. However, the roles of miRNAs in multiple biological processes or various diseases and their underlying molecular mechanisms still have not been fully understood yet. Predicting potential miRNA-disease associations by integrating various heterogeneous biological datasets is of great significance to the biomedical research. Computational methods could obtain potential miRNA-disease associations in a short time, which significantly reduce the experimental time and cost. Considering the limitations in previous computational methods, we developed the model of Within and Between Score for MiRNA-Disease Association prediction (WBSMDA) to predict potential miRNAs associated with various complex diseases. WBSMDA could be applied to the diseases without any known related miRNAs. The AUC of 0.8031 based on Leave-one-out cross validation has demonstrated its reliable performance. WBSMDA was further applied to Colon Neoplasms, Prostate Neoplasms, and Lymphoma for the identification of their potential related miRNAs. As a result, 90%, 84%, and 80% of predicted miRNA-disease pairs in the top 50 prediction list for these three diseases have been confirmed by recent experimental literatures, respectively. It is anticipated that WBSMDA would be a useful resource for potential miRNA-disease association identification.

MicroRNAs (miRNAs) are one kind of endogenous non-coding RNAs (ncRNAs) with the length of 20 ~ 25 nucleotides. They could bind to the 3′ untranslated regions (UTRs) and suppress the expression of their target messenger RNAs (mRNAs) at post-transcriptional level through sequence-specific base pairing^1^,^2^,^3^,^4^. However, some studies have reported that miRNAs could also function as positive regulators^5^,^6^. Until now, thousands of miRNAs have been discovered in the eukaryotic organisms ranging from nematodes to humans based on various experimental methods and computational models^7^,^8^.

Accumulating studies have shown that miRNAs play a critical role in many important biological processes, including cell proliferation^9^, development^10^, differentiation^11^, and apoptosis^12^, metabolism^13^,^14^, aging^13^,^14^, signal transduction^15^, viral infection^11^ and so on. In particular, it was observed that miRNAs with similar sequences or secondary structures tend to play roles in similar biological processes^16^. Furthermore, the dysregulations of the miRNAs have been confirmed to be associated with the development and progression of various complex human diseases^17^,^18^,^19^. Recent plenty of studies have found that miRNAs are associated with various cancers or cancer related processes^20^. For example, mir-335 and mir-31 are considered to be the robust inhibitors in breast cancer^21^,^22^,^23^. Another example is mir-21, whose upregulation could promote hormone-dependent and hormone-independent growth in prostate cancer^24^,^25^. What’s more, mir-101 was found to be involved in human breast cancer by targeting Stathmin1, and mir-185 was found to be involved in human breast carcinogenesis by targeting Vegfa^26^,^27^. The levels of mir-27b and miR-134 were found significantly lower in lung tumors than normal tissue, which suggested that they are associated with lung cancer^28^. Identifying disease-related miRNAs could benefit disease diagnosis, treatment, and prevention^29^,^30^,^31^. However, using experimental methods to identify the associations between miRNAs and diseases is demanding and costly. As more and more biological datasets are available, it would be an effective way to develop computational methods to uncover the potential associations between miRNAs and diseases^32^,^33^,^34^,^35^,^36^,^37^,^38^,^39^.

In the past few years, significant progresses have been made in potential miRNA-disease association identification. Various computational methods have been developed from network and systems biology points of view in recent years, which could be further divided into the similarity measure-based approaches and machine learning-based approaches. Furthermore, most of computational methods were developed based on the assumption that functionally similar miRNAs usually have connection with phenotypically similar diseases^40^,^41^,^42^.

By integrating miRNA functional interactions, disease phenotype similarities, and known miRNA-disease associations, Jiang *et al.*^30^ developed a hypergeometric distribution-based computational model that prioritized the entire microRNAome for the investigated diseases to predict potential disease-associated miRNAs. This computational model strongly relies on predicted miRNA-target interactions which have a high rate of false-positive and high false-negative results. Furthermore, Xuan *et al.*^43^ proposed a method called HDMP based on weighted k most similar neighbors to predict disease-related miRNA candidates. They calculated the functional similarity between miRNAs from the information content of disease terms and phenotype similarity between diseases and considered the miRNA family and the cluster information to recalculate miRNA functional similarity by assigning higher weight to members of miRNA family or cluster. However, the determination of the value of the number of neighbors will have a great influence on the performance of HDMP. Local network similarity measure has been adopted in above two studies, which only considered miRNA neighbor information in the scoring system. In recent studies, global network similarity measure has demonstrated their more reliable performance than local network similarity measure-based ones^44^,^45^,^46^. Based on the assumption that global network similarity measures are better than traditional local network similarity measures in uncovering potential associations between diseases and miRNAs, Chen *et al.*^37^ developed the model of Random Walk with Restart for MiRNA–Disease Association (RWRMDA) to infer potential miRNA–disease interaction by implementing random walk on the miRNA functional similarity network, which didn’t rely on the predicted miRNA-target interactions. RWRMDA has obtained a good predictive accuracy, but this model is not applicable for diseases without any known associated miRNAs.

According to the assumption that if miRNAs are implicated in a specific tumor phenotype, their target genes will be aberrantly regulated, Xu, *et al.*^47^ constructed a heterogeneous miRNA-target dysregulated network, extracted four network topological features, and developed Support Vector Machine (SVM)-based Supervised classifier to distinguish positive disease related miRNAs from negative ones. However, it is difficult and even impossible to obtain negative disease-related miRNAs. Based on the framework of regularized least squares, Chen *et al.*^35^ further proposed a semi-supervised method Regularized Least Squares for MiRNA-Disease Association (RLSMDA) by integrating disease-disease semantic similarity network, miRNA-miRNA functional similarity network, and known human miRNA-disease associations. RLSMDA does not need negative samples and could be effectively applied to diseases without any known related miRNAs.

Other computational models tried to predict miRNA-disease associations based on known disease-related genes and predicted miRNA-target interactions. For example, Shi *et al.*^48^ proposed a computational method to predict miRNA-disease associations by focusing on the functional link between miRNA targets and disease genes in protein-protein networks. Mørk *et al.*^49^ proposed a method called miRPD to predict potential miRNA-disease associations by integrating miRNA-protein associations and protein-disease interactions text mined from the literature. Xu *et al.*^50^ presented a miRNA prioritization approach by using the functional similarities between miRNA target genes derived from matched miRNA and mRNA expression dataset and known disease genes. However, the molecular bases for only less than 40% of human diseases are partly known and we can’t obtain highly accurate miRNA-target interactions, which have limited the application of these methods.

As mentioned above, the exiting methods have different limitations. For example, miRNA-target interactions and disease-genes associations used in some methods are incomplete or inaccurate. Furthermore, many methods couldn’t be applied to disease without any known related miRNAs. Therefore, new effective computational methods are urgently in need. Based on the assumption that functional similar miRNAs tend to interact with similar diseases, we developed the model of Within and Between Score for MiRNA-Disease Association prediction (WBSMDA) by integrating known miRNA-disease associations, miRNA functional similarity network, disease semantic similarity network, and Gaussian interaction profile kernel similarity network to uncover the potential disease-miRNA associations. WBSMDA is applicable for diseases without any known related miRNAs. LOOCV was implemented for WBSMDA and the AUC of 0.8031 has been obtained, which demonstrated the reliable and effective performance of WBSMDA. Then, WBSMDA was evaluated by the case studies of Colon Neoplasms, Prostate Neoplasms and lymphoma. As a result, 45, 40 and 42 out of top 50 predicted miRNA-disease associations for these three important diseases were confirmed by recent experimental literatures, respectively.

## Results

### Leave-one-out cross validation

**Figure 1:**
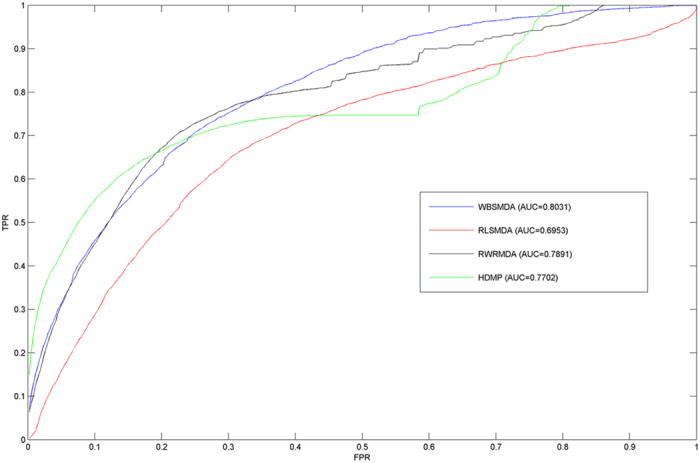
The comparison result between WBSMDA and these three methods was shown, which demonstrated the superiority performance of WBSMDA to previous computational models.

LOOCV was implemented on known miRNA-disease associations obtained from HMDD^51^ to evaluate the predictive performance of WBSMDA. For each given disease *d*, each known disease-related miRNA was left out in turn as test miRNA and other known disease-related miRNAs were taken as training miRNAs. All miRNAs without known evidences to be associated with the disease *d* were selected to be candidate miRNAs. Then we can get the rank of this test miRNA among the candidate miRNAs. If the rank exceeds the given threshold, the WBSMDA model was considered to have made a correct prediction of this miRNA-disease association. Receiver-Operating Characteristics (ROC) curve was drawn by plotting true positive rate (TPR, sensitivity) versus false positive rate (FPR, 1-specificity) at different thresholds. Here, Sensitivity refers to the percentage of the test miRNA-disease associations which are ranked higher than the given threshold. And specificity (also called the true negative rate) refers to the percentage of negative miRNA-disease pairs below the threshold. When we vary the thresholds of successful prediction, we can obtain the corresponding TPR and FPR. In this way, ROC could be drawn and the area under ROC curve (AUC) could be calculated to evaluate the performance of WBSMDA. If AUC = 1, it means that the WBSMDA has perfect performance. And AUC = 0.5 indicates random performance. As a result, WBSMDA achieved a reliable AUC of 0.8031 (See Fig. 1).

### Compared with other methods

We further compared WBSMDA with the following three classical methods which have been confirmed to achieve excellent prediction accuracy based on the previous version of known miRNA-disease associations in HMDD^51^: 1)RLSMDA^35^, which predicted disease-related miRNAs based on the framework of regularized least squares; 2)RWRMDA^37^, which implemented random walk on the miRNA functional similarity network to predict novel miRNA-disease associations; 3)HDMP^43^, which predicted potential disease-related miRNAs based on weighted k most similar neighbors. The comparison result between WBSMDA and these three methods was shown in Fig. 1, which demonstrated the superiority performance of WBSMDA to previous computational models. Especially, WBSMDA significantly improved the performance of RLSMDA with the AUC increase of 0.11. RWRMDA and HDMP can’t be used to diseases without any known associated miRNAs and miRNAs without any known related diseases. Therefore, except for performance improvement over these two computational models, WBSMDA could effectively overcome this important limitation.

Furthermore, we implemented 5-fold cross validation for miRNA-disease association prediction evaluation. All the known miRNA-disease associations have been divided into 5 groups with equal sizes, where 4 groups would be regarded as training samples for model learning and the other group would be used for model evaluation. We implemented 100 randomized divisions of known associations to minimize the performance difference resulting from samples divisions. As a result, WBSMDA has obtained the reliable performance (the mean and the standard deviation of AUCs is 0.8185 and 0.0009, respectively.).

### Case studies

WBSMDA was applied to predict potential miRNA-disease associations for all the diseases investigated in this paper. To further demonstrate the prediction ability of WBSMDA, case studies of Colon Neoplasms, Lymphoma and Prostate Neoplasms were implemented here. The prediction results were validated based on another two important miRNA-disease association databases, miR2Disease^52^ and dbDEMC database^53^. One important fact must be pointed out is that only the associations which are not recorded in the HMDD database would be regarded as validation datasets. Therefore, validation datasets is totally independent of datasets used for prediction.

Colon Neoplasms (CN) are a big threaten to people’s lives with a low detection rate at early stages^54^,^55^. There is an increasing need of novel sensitive biomarkers that could help improve the detection of CN^56^. For example, miRNA hsa-mir-145 can inhabits the growth of CN cells by targeting the insulin receptor substrate-1, and hsa-mir-126 could suppress the growth of CN cells by targeting phosphatidylinositol 3-kinase signaling^57^,^58^. Taking CN as a case study, WBSMDA was implemented to prioritize candidate miRNAs (See Table 1 and Supplementary Table 1). As a result, nine of the top ten potential related miRNAs were confirmed to be associated with CN. Furthermore, forty-five out of top fifty potential CN-associated miRNAs predicted by WBSMDA were confirmed to be associated with CN. Among those predicted CN-associated miRNAs, hsa-mir-20a (1st in the prediction list) was confirmed to up-regulated in three or more types of solid cancers, including CN^24^. Studies have found mir-18a (2nd in the prediction list) may function as a tumor suppressor by targeting K-Ras in CN^59^. What’s more, hsa-mir-19b and hsa-mir-19a (3rd and 4th in the prediction list, respectively) were confirmed to be differentially expressed between CN and normal colorectal tissue^60^.

Lymphoma could be divided to two main categories: Hodgkin lymphomas (HL) and the non-Hodgkin lymphomas (NHL). HL is more frequently occurring lymphatic cancer with three to four novel cases per 100,000 individuals every year in the Western population. Furthermore, HL is difficult to be diagnosed at early stages^61^,^62^. NHL is a heterogeneous group of malignancies that originate in lymphatic hematopoietic tissue. NHL is treated mainly through chemotherapy treatment and local radiotherapy and could be further classified into B-cell lymphomas and T-cell lymphomas^63^. Recent experimental studies showed that the down-regulation of mir-16, mir-101 and mir-138 in the t (14;18)-negative FL (follicular lymphoma) subset was connected to profound mRNA expression changes of potential target genes involving cell cycle control and apoptosis^64^. MiRNA hsa-mir-19a showed an increased expression level compared with normal canine peripheral blood mononuclear cells (PBMC) and normal lymph nodes (LN) in canine B-cell lymphomas^65^. Taking lymphomas as a case study to implement WBSMDA for potential miRNA-disease association prediction, top ten potential lymphoma-associated miRNAs in the prediction list were all successfully verified based on recent experimental reports (See Table 1 and Supplementary Table 2). Furthermore, for the top fifty predicted lymphoma-associated miRNAs predicted by WBSMDA, forty-two of them have experimental literature evidences. For example, the up-regulation of miRNA hsa-mir-183 (1st in the prediction list), hsa-mir-215(2nd in the prediction list), hsa-mir-9 (3rd in the prediction list), hsa-mir-34a (5th in the prediction list) and down-regulation of hsa-mir-30b (4th in the prediction list) are all related to the development of lymphoma.

Prostate Neoplasms (PN) is the second leading cause of cancer-related death among men in developed countries^66^,^67^. About 29,720 patients died of PN in 2013 in the USA and it is estimated that there will be about 220,800 new cases in 2015^66^,^67^,^68^. The initial treatment for most patients with PN is generally effective, while then PN will progresses to CRPC (castration-resistant prostate cancer) which is difficult to treat^66^. MiRNA mir-145 was deregulated in PN by targeting the proto-oncogene ERG^69^. It was also reported that androgen represses the mir-99a/let7c/125b-2 cluster through androgen receptor (AR) which can stimulate and repress gene expression to promote the initiation and progression of PN^70^. Taking PN as a case study to implement WBSMDA, eight predicted PN-associated miRNAs of the top ten prediction list and forty of top fifty prediction list were verified based on experimental reports (See Table 1 and Supplementary Table 3). For example, the expression of hsa-mir-143 (1st in the prediction list) and hsa-mir-199a (4th in the prediction list) is different in PN compared with the benign prostatic hyperplasia samples^71^. Studies also found that miRNA hsa-mir-126 (2nd in the prediction list) was one of the upregulated miRNAs in PN with perineural invasion (FDR 10%)^72^. Ectopic has-mir-34a (4th in the prediction list) expression could induce apoptosis of PN cells, and could result in cell cycle arrest, growth inhibition and attenuated chemoresistance to anticancer drug camptothecin, suggesting that has-mir-34a could sever as a potential choice for the treatment of p53-defective PN^73^.

## Discussions

As increasing evidences indicated that miRNAs are closely related to the development and progression of different kinds of human diseases, more and more attentions have been focused on the identification of novel miRNA-disease associations. Developing computational methods to predict novel miRNA-disease associations have attracted a lot of attentions because they could effectively decrease the time and cost of biological experiments by quantifying the miRNA-disease association probability and selecting the associations with high scores for further experimental validation. In this paper, we developed a novel computational model of WBSMDA to predict potential miRNA-disease associations by integrating known miRNA-disease associations derived from HMDD, miRNA functional similarity network, disease semantic similarity network, and Gaussian interaction profile kernel similarity for diseases and miRNA. WBSMDA obtained a reliable AUC of 0.8031 in the validation schema of LOOCV, demonstrating the superior performance to previous classical computational models. Furthermore, case studies of Colon Neoplasms, lymphoma and Prostate Neoplasms were implemented and 90%, 84%, and 80% of predicted miRNA-disease pairs in the top 50 prediction list for these three important diseases have been confirmed based on recent experimental literatures, respectively. It is anticipated that WBSMDA could be an important and useful miRNA-disease association prediction computational model with the potential value for human disease diagnosis, treatment, prognosis, and prevention.

In conclusion, the reliable performance of WBSMDA could be further attributed to the following factors, which also constitute the novelty of WBSMDA. Firstly, we obtained known experimentally confirmed miRNA-disease associations from highly reliable HMDD database and used them as the seed samples to predict potential associations between miRNAs and diseases. Then, plenty of heterogeneous biological datasets were integrated in WBSMDA, including known miRNA-disease associations, miRNA functional similarity network, disease semantic similarity network, and Gaussian interaction profile kernel similarity, which benefit the improvement of prediction accuracy and decrease the prediction bias. Furthermore, new diseases (diseases without any known related miRNAs) and new miRNAs (miRNAs without any known associated diseases) have been discovered each year. Therefore, it is very important to design novel and effective computational models for new diseases and miRNAs. WBSMDA could work for diseases without any known related miRNAs and miRNAs without any known associated diseases by quantifying the association probability between each candidate miRNA-disease pair and selecting the most promising associations for experimental validation, overcoming the limitations of most of previous computational models. Finally, as a global ranking model, WBSMDA could predict miRNA-disease association for all diseases simultaneously.

Of course, WBSMDA also have some limitations that need to be improved in the future. Firstly, since WBSMDA is developed based on the known miRNA-disease associations with the assumption that functional similar miRNAs are more likely to have connection with phenotypically similar diseases, it may cause bias to miRNAs with more known associated diseases. Furthermore, although WBSMDA has significantly improved previous methods, current predictive accuracy is still not very satisfactory based on the evaluation of LOOCV. In the future, the prediction performance of WBSMDA will be further improved by integrating more reliable biological datasets and obtaining more known miRNA-disease associations. Finally, how to more reasonably integrate similarity measure and integrate Within-Score and Between-Score to calculate the association score of miRNA-disease pair deserve further research in the future.

## Methods

### Human miRNA-disease associations

Human miRNA-disease associations were downloaded from the latest version of HMDD database, including 5430 experimentally verified human miRNA-diseases associations about 383 diseases and 495 miRNAs (see Supplementary Table 4). To better describe the miRNAs-disease associations, we use the adjacency matrix ***A***, in which the entity *A(i,j)* is 1 if miRNA *m(j)* is confirmed to be related to disease *d(i)*, otherwise 0. Furthermore, variable *nm* and *nd* denotes the number of miRNAs and diseases investigated in this study, respectively.

### MiRNA functional similarity

In previous work^74^, miRNA functional similarity score was calculated based on the assumption that functionally similar miRNAs tend to be associated with phenotypically similar diseases. We downloaded miRNA functional similarity scores from http://www.cuilab.cn/files/images/cuilab/misim.zip in January 2010. Similarly, miRNA functional similarity matrix *FS* was constructed, where the entity *FS(m(i), m(j))* represents the functional similarity between miRNA *m(i)*and *m(j)*.

### Disease semantic similarity

Each disease can be described as a Directed Acyclic Graph (DAG) and *DAG(D)* = *(D,T(D),E(D))* was used to represent the disease *D*, where *T(D)* is the node set including node *D* itself and its ancestor nodes, *E(D)* is the corresponding edge set including the direct edges from parent nodes to child nodes^74^. The semantic value of disease *D* could be calculated as follows:


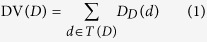



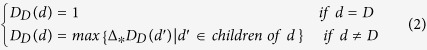


where 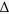 is the semantic contribution factor. For disease *D*, the contribution of itself to the semantic value of disease *D* is 1 and the contribution decrease as the distance between *D* and other disease increases. Therefore, disease terms in the same layer would have the same contribution to the semantic value of disease *D*.

Based on the assumption that two diseases sharing larger part of their DAGs are considered to have larger semantic similarity, the semantic similarity between disease 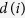 and 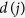 can be defined as follows:


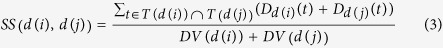


where 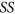 is the disease semantic similarity matrix.

### Gaussian interaction profile kernel similarity for diseases

Based on the assumption that functional similar miRNAs tend to be associated with similar diseases, Gaussian interaction profile kernel similarity for diseases are calculated by considering the topologic information of known miRNA–disease association network. Firstly, we used binary vector *IP(d(i))* to denote the interaction profiles of disease *d(i)* by observing whether disease *d(i)* is associated with each miRNA or not, i.e. the *i*th row of the adjacency matrix *A*. Then, Gaussian kernel similarity between disease *d(i)* and *d(j)* was defined based on their interaction profiles as follows.


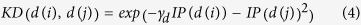


where parameter 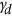 was used to control the kernel bandwidth and obtained by normalizing a new bandwidth parameter 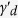 by the average number of associations with miRNAs for all the diseases.

Therefore, 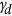 was defined as follows.


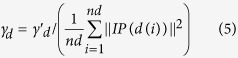


Finally, *KD* is the Gaussian interaction profile kernel similarity matrix for diseases, where the entity *KD(*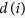,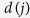) is the Gaussian interaction profile kernel similarity between disease *d(i)* and *d(j)*.

### Gaussian interaction profile kernel similarity for miRNAs

Similar to disease Gaussian interaction profile kernel similarity calculation, miRNA Gaussian interaction profile kernel similarity matrix can be calculated in a similar way:


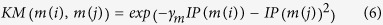



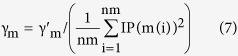


Here, interaction profile 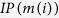 of miRNA 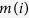 was defined to denote whether 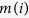 is associated with each disease or not. 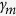 was obtained through the normalization of a new bandwidth parameter 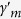 by the average number of associated diseases for all the miRNAs.

### Integrated similarity for miRNAs and diseases

Here, integrated miRNA similarity matrix 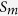 and integrated disease similarity matrix 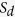 were constructed based on miRNA functional similarity, disease semantic similarity, and Gaussian interaction profile kernel similarity, respectively.


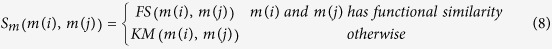



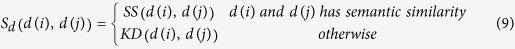


### WBSMDA

**Figure 2:**
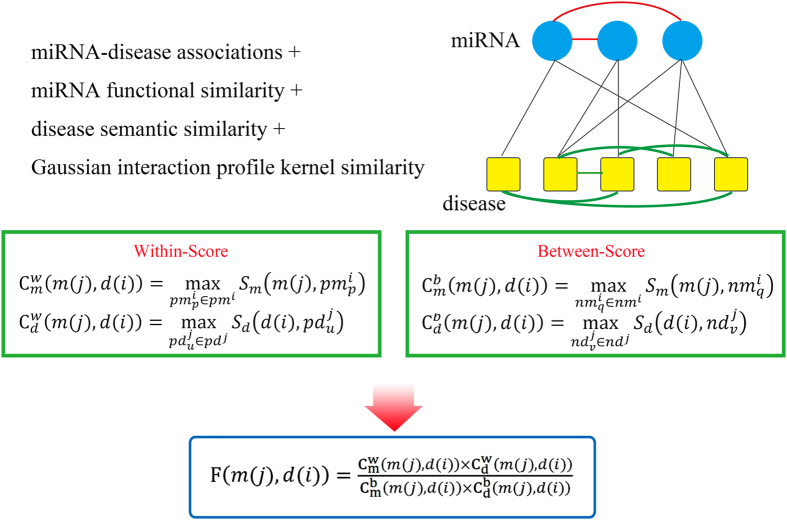
Flow chart of WBSMDA demonstrating the basic ideas of predicting potential disease-related miRNAs by integrating known miRNA-disease associations, miRNA functional similarity, disease semantic similarity, and Gaussian interaction profile kernel similarity. Within-Score and Between-Score were calculated and combined to obtain the final score for potential miRNA-disease association inference.

Based on the assumption that functional similar miRNAs tend to be associated with similar diseases and vice versa, we developed the method of Within and Between Score for MiRNA-Disease Association prediction (WBSMDA) to predict potential miRNA-disease associations (see Fig. 2, motivated by literature^75^). Within-Scores and Between-Scores for miRNA-disease pair 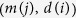 were defined as follows:


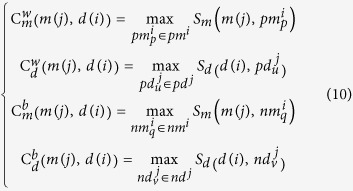


where 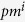 was the miRNA group that has known relation with disease 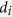, 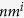 was the miRNA group that does not have known relation with disease 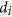, 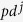 was the disease group which is associated with miRNA 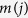 in the known miRNA-disease association dataset, and 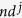 was the disease group which is not proved to be associated with miRNA 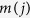 in the known miRNA-disease association dataset. Briefly speaking, from the view of miRNA, the Within-Score is to find the miRNA that has the highest similarity score with investigated miRNA among the group of miRNAs with known association with the investigated disease. The Between-Score is to find the miRNA that has the highest similarity score with investigated miRNA in the group of miRNAs without known association with the investigated disease. Also from the view of disease, the Within-Score and Between-Score were defined in the same way.

Here, we combined Within-Score and Between-Score from the view of miRNA and diseases to calculate the association probability for miRNA-disease pair 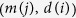 as follows:


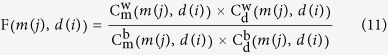


Furthermore, for new diseases *d* without any known related miRNAs, we could integrate Within-Score and Between-Score from the view of diseases to predict its related miRNAs as follows:


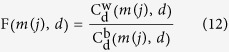


Also, for new miRNAs *m* without any known associated diseases, Within-Score and Between-Score from the view of miRNAs could be integrated to predict its potential associated diseases as follows:


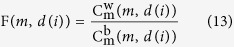


## Additional Information

**How to cite this article**: Chen, X. *et al.* WBSMDA: Within and Between Score for MiRNA-Disease Association prediction. *Sci. Rep.* **6**, 21106; doi: 10.1038/srep21106 (2016).

## Supplementary Material

Supplementary Information

Supplementary Table 1

Supplementary Table 2

Supplementary Table 3

Supplementary Table 4

## Figures and Tables

**Table 1:** WBSMDA was applied to Colon Neoplasms, lymphoma, Prostate Neoplasms to identify their potential associated miRNAs. As a result, 9, 10, and 8 of top 10 predicted pairs for these diseases have been confirmed based on recent experimental literatures.

miRNA	Disease	Association score	Evidence
hsa-mir-20a	Colon Neoplasms	0.9442	dbdemc;miR2Disease
hsa-mir-18a	Colon Neoplasms	0.8654	miR2Disease
hsa-mir-19b	Colon Neoplasms	0.8581	dbdemc;miR2Disease
hsa-mir-19a	Colon Neoplasms	0.8552	dbdemc;miR2Disease
hsa-mir-143	Colon Neoplasms	0.8005	dbdemc;miR2Disease
hsa-mir-92a	Colon Neoplasms	0.7484	unconfirmed
hsa-mir-191	Colon Neoplasms	0.7319	dbdemc;miR2Disease
hsa-mir-132	Colon Neoplasms	0.7166	miR2Disease
hsa-mir-29b	Colon Neoplasms	0.6982	dbdemc;miR2Disease
hsa-mir-34a	Colon Neoplasms	0.6755	dbdemc;miR2Disease
hsa-mir-183	lymphoma	0.3882	dbdemc
hsa-mir-215	lymphoma	0.382509	dbdemc
hsa-mir-9	lymphoma	0.377564	dbdemc
hsa-mir-30b	lymphoma	0.375303	dbdemc
hsa-mir-34a	lymphoma	0.367483	dbdemc
hsa-let-7a	lymphoma	0.364527	dbdemc
hsa-mir-145	lymphoma	0.364476	dbdemc;miR2Disease
hsa-mir-205	lymphoma	0.358745	dbdemc
hsa-mir-106b	lymphoma	0.355309	dbdemc
hsa-mir-106a	lymphoma	0.353891	dbdemc;miR2Disease
hsa-mir-143	Prostate Neoplasms	0.8005	dbdemc;miR2Disease
hsa-mir-126	Prostate Neoplasms	0.7654	dbdemc;miR2Disease
hsa-mir-203	Prostate Neoplasms	0.7117	unconfirmed
hsa-mir-199a	Prostate Neoplasms	0.7089	dbdemc;miR2Disease
hsa-mir-34a	Prostate Neoplasms	0.6755	dbdemc;miR2Disease
hsa-mir-200b	Prostate Neoplasms	0.6695	unconfirmed
hsa-mir-127	Prostate Neoplasms	0.6642	dbdemc;miR2Disease
hsa-mir-141	Prostate Neoplasms	0.6609	mi2Disease
hsa-mir-194	Prostate Neoplasms	0.6571	dbdemc;miR2Disease
hsa-mir-223	Prostate Neoplasms	0.645	dbdemc;miR2Disease
