# Supplementary material for: WBSMDA: Within and Between Score for MiRNA-Disease Association prediction
Source: Sci Rep. 2016 Feb 16;6:21106. doi: 10.1038/srep21106 (PMC4754743; doi:10.1038/srep21106)
Supplement: Supplementary Information [file srep21106-s1.doc]

**WBSMDA:** **Within and Between Score for MiRNA-Disease Association prediction**

Xing Chen^1,2,*,#^, Chenggang Clarence Yan^3,4,#^, Xu Zhang^5^, Zhu-Hong You^6^, Lixi Deng^7,8^, Ying Liu^9, *^, Yongdong Zhang^10^, Qionghai Dai^4^

^1^National Center for Mathematics and Interdisciplinary Sciences,

Chinese Academy of Sciences, Beijing, 100190, China

^2^Academy of Mathematics and Systems Science,

Chinese Academy of Sciences, Beijing, 100190, China

^3^Institute of Information and Control,

Hangzhou Dianzi University, Hangzhou, 310018, China

^4^Department of Automation, Tsinghua University, Beijing, 100084, China

^5^School of Mechanical, Electrical & Information Engineering,

Shandong University, Weihai, 264209, China

^6^School of Computer Science and Technology,

China University of Mining and Technology, Xuzhou, 221116, China

^7^ Institute of Computing Technology,

Chinese Academy of Sciences, Beijing, 100190, China

^8^University of Chinese Academy of Sciences, Beijing, 100049, China

^9^School of Economics and Management, Beihang University, Beijing, 100191, China

^10^Key Lab of Intelligent Information Processing of Chinese Academy of Sciences, Institute of Computing Technology, Chinese Academy of Sciences,

Beijing, 100190, China

*Corresponding authors

#The authors wish it to be known that, in their opinion, the first two authors should be regarded as joint First Authors.

**Email**: [xingchen@amss.ac.cn](mailto:xingchen@amss.ac.cn); [Liuying@buaa.edu.cn](mailto:Liuying@buaa.edu.cn)

**Supplementary Information**

**Supplementary Table 1.** Taking Colon Neoplasms (CN) as a case study, WBSMDA was implemented to prioritize candidate miRNAs. As a result, nine of the top ten potential related miRNAs have been confirmed to be associated with CN. Furthermore, forty-five out of top fifty potential CN-associated miRNAs predicted by WBSMDA were confirmed to be associated with CN.

**Supplementary Table 2.** Taking lymphomas as a case study to implement WBSMDA for potential miRNA-disease association prediction, top ten potential lymphoma-associated miRNAs in the prediction list were all successfully verified based on recent experimental reports. Furthermore, for the top fifty predicted lymphoma-associated miRNAs predicted by WBSMDA, forty-two of them have experimental literature evidences.

**Supplementary Table 3.** Taking Prostate Neoplasms (PN) as a case study to implement WBSMDA, eight predicted PN-associated miRNAs of the top ten prediction list and forty of top fifty prediction list were verified based on experimental reports.

**Supplementary Table 4.** Human miRNA-disease associations were downloaded from the latest version of HMDD database, including 5430 experimentally verified human miRNA-diseases associations about 383 diseases and 495 miRNAs.
